# Syringe plunger: An alternative sterile skin-marker in plastic surgery

**DOI:** 10.1016/j.jpra.2019.01.006

**Published:** 2019-01-12

**Authors:** Fabrizio Schönauer, Rosita Pensato, Francesco D'Andrea, Giovanni Francesco Nicoletti

**Affiliations:** a Federico II, Department Plastic and Reconstructive Surgery, University of Naples, via Pansini 5, 80131 Naples, Italy; b Multidisciplinary Department of Medical-Surgical and Dental Specialties, Plastic Surgery Unit, University of Study of Campania “Luigi Vanvitelli”, piazza Miraglia, 80138, Naples, Italy

**Keywords:** Skin-marker, Ink, Intraoperative, Syringe plunger, Sterile

Dear Sir,

Surgical planning is mandatory in plastic surgery. First, Tagliacozzi, in the 19th century used a thin plate of wax fitted on the stump of the nose, then flattened and laid out on the forehead; the drawing was done around the wax. At that time Gentian violet was the only ink available; gentian violet is a greenish-bronze, odorless crystal that is poorly soluble in water but highly soluble in alcohol. It is available for medical use. Today it is the most used agent in trade for surgical marking pens.

Bankoff,^1^ in 1943, recommended the use of methylene blue for beginner surgeons only. This ink is a dark-green odorless hygroscopic crystal and moderately soluble in water and ethyl alcohol. It is used for tracing fistulae, sinus tracts, skin incision and to colour saline solution in tissue-expanders. After the creation of the first aniline dye in 1856 by Perkins,^1^ hundreds of dyes and stains were developed.

Brilliant green (1877), is a gold-coloured crystal, which is highly soluble in water and ethyl alcohol. It is also used as ink and for microbiological testing. Victor Bonney.^1^ in 1915 realized his ‘violet green antiseptic’ (Bonney's blue), which contained both gentian violet and brilliant green.

Sommerlad.^2^ in 1973 described a new instrument: a single stainless-steel rod with a four-point tip to dip in the ink (Bonney's Blue) and use in a sterile environment. This is the gold-standard for intraoperative drawing, but it is not available in all surgical instrument catalogues. Dellatorre and Bochnia Cerci.^3^ in 2015 described the syringe pen, a handmade device that consists of a syringe full of Methylene blue with a toothpick inside the tip that can be dipped in ink. Although it is less expensive than other marking pens, wood can release splinters with a loss of precision in drawing lines.

Preoperative use of unsterile skin markers (Sharpie W10 black, Dual Tip, Easimark modern regular tip etc.) exposes to cross infection risk.^4^ Another disadvantage is the possible erasing of the drawing during surgical prep. On the other side single-use pens are not always available and they are more expensive than the above mentioned markers^5^.

Our skin-marker, on the contrary, consists of the plunger of an insulin syringe (Insulight®). It is fitted with a rubber stopper with a truncated cone shape that matches the needle hub. (Figure 1). It is immediately ready to use and already sterile. It can be dipped in sterile Bonney's Blue ink and used for intraoperative drawing. Other advantages are that it is inexpensive, easy to handle and results in a proper ink distribution without being a vehicle of infection. This versatile device has been used several times to intraoperatively draw (Figure 2) when Sommerlad pen was unavailable for surgery planning.

**Figure 1:**
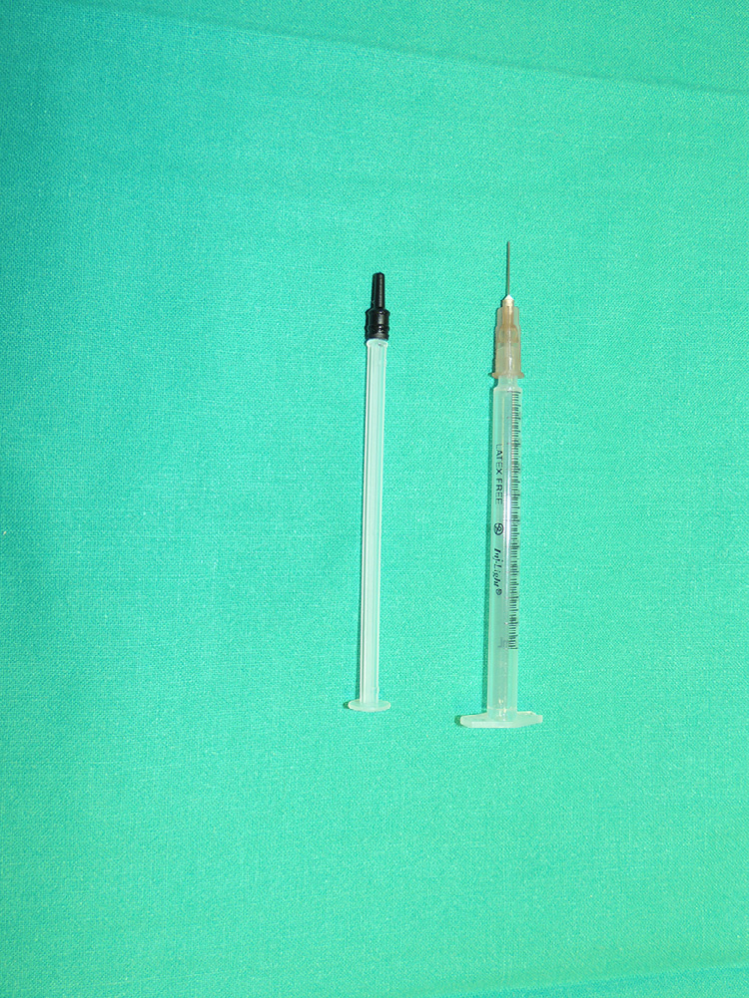
Syringe plunger (Insulight ®).

**Figure 2:**
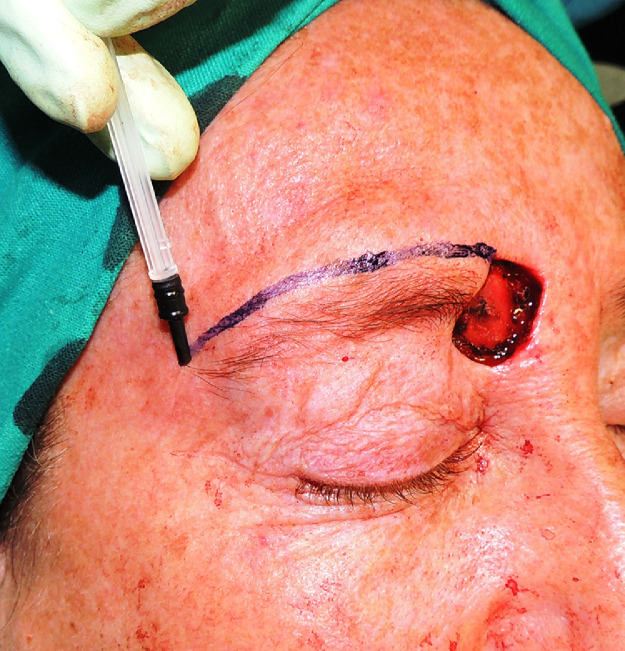
Intraoperative use of the syringe plunger as skin marker in flap planning.

## Funding sources

None.

## IRB approval status

Not needed.

## Conflicts of Interest

None declared.
